# Yeast pentatricopeptide protein Dmr1 (Ccm1) binds a repetitive AU-rich motif in the small subunit mitochondrial ribosomal RNA

**DOI:** 10.1261/rna.074880.120

**Published:** 2020-09

**Authors:** Jakub Piątkowski, Paweł Golik

**Affiliations:** 1 Institute of Genetics and Biotechnology, Faculty of Biology, University of Warsaw, Warsaw, 02-106, Poland; 2 Institute of Biochemistry and Biophysics, Polish Academy of Sciences, Warsaw, 02-106, Poland

**Keywords:** mitochondria, mitoribosome, pentatricopeptide protein, rRNA, yeast

## Abstract

PPR proteins are a diverse family of RNA binding factors found in all Eukaryotic lineages. They perform multiple functions in the expression of organellar genes, mostly on the post-transcriptional level. PPR proteins are also significant determinants of evolutionary nucleo-organellar compatibility. Plant PPR proteins recognize their RNA substrates using a simple modular code. No target sequences recognized by animal or yeast PPR proteins were identified prior to the present study, making it impossible to assess whether this plant PPR code is conserved in other organisms. Dmr1p (Ccm1p, Ygr150cp) is a *S. cerevisiae* PPR protein essential for mitochondrial gene expression and involved in the stability of 15S ribosomal RNA. We demonstrate that in vitro Dmr1p specifically binds a motif composed of multiple AUA repeats occurring twice in the 15S rRNA sequence as the minimal 14 nt (AUA)_4_AU or longer (AUA)_7_ variant. Short RNA fragments containing this motif are protected by Dmr1p from exoribonucleolytic activity in vitro. Presence of the identified motif in mtDNA of different yeast species correlates with the compatibility between their Dmr1p orthologs and *S. cerevisiae* mtDNA. RNA recognition by Dmr1p is likely based on a rudimentary form of a PPR code specifying U at every third position, and depends on other factors, like RNA structure.

## INTRODUCTION

The current widely accepted hypothesis explaining the evolutionary origin of the complex internal structure of Eukaryotic cells points to an endosymbiotic origin of mitochondria and chloroplasts, that still possess vestigial genomes of eubacterial origin (Gray 1999, 2012; Pittis and Gabaldón 2016). Organellar genomes show great evolutionary diversity, but they share some common features of gene expression, including polycistronic transcription units, simplified regulation of transcription initiation, and reliance on complex post-transcriptional mechanisms of RNA processing, stability, and translational control.

Pentatricopeptide (PPR) proteins are a prominent family of mostly organellar RNA-binding proteins, common to all eukaryotes, and involved in various aspects of RNA metabolism (Rackham and Filipovska 2012; Giegé 2013; Herbert et al. 2013; Lightowlers and Chrzanowska-Lightowlers 2013). The PPR proteins are α-solenoid proteins composed mostly of multiple tandem repeats of a degenerate 35-amino acid motif consisting of two antiparallel α-helices (Filipovska and Rackham 2013). They are remarkably abundant in land plants, where they constitute the most numerous paralogous families, with as many as several hundreds of members encoded in a single genome (Small and Peeters 2000; O'Toole et al. 2008; Giegé 2013).

Sequence-specific binding of RNAs is postulated for all the PPR proteins, but exact target sequences and mechanisms of substrate recognition were identified only for several plant members of the family (Meierhoff et al. 2003; Okuda et al. 2006; Pfalz et al. 2009; Barkan et al. 2012; Okuda and Shikanai 2012; Ban et al. 2013; Haïli et al. 2013; Ke et al. 2013; Yagi et al. 2013; Yin et al. 2013). Bioinformatic and structural studies revealed that these proteins recognize their target sequences in a modular fashion, wherein the amino acid residues at two to three positions in the two α-helices of a PPR motif recognize a single nucleotide of the RNA substrate (Barkan et al. 2012; Ban et al. 2013; Ke et al. 2013; Takenaka et al. 2013; Yagi et al. 2013; Yin et al. 2013). The deterministic nature of this recognition code makes designing synthetic PPR proteins targeted at arbitrarily chosen RNA sequences possible (Filipovska and Rackham 2013; Coquille et al. 2014; Gully et al. 2015), as well as allows for prediction of target RNA based on protein sequence alone (Harrison et al. 2016; Yan et al. 2019).

Genetic and molecular studies identified RNA molecules that are targets of the seven human PPR proteins (for review, see Lightowlers and Chrzanowska-Lightowlers 2013), as well as the majority of yeast members of the family (for review, see Herbert et al. 2013). Similarly, many PPR proteins of trypanosomes were found to be involved in mitochondrial ribosome function and translational control (Pusnik et al. 2007; Aphasizhev and Aphasizheva 2013; Aphasizheva et al. 2016). In *Trypanosoma brucei*, two PPR proteins were found to bind poly(A) and poly(G) stretches in mitochondrial RNAs (Kamba et al. 2018; Mesitov et al. 2019). In spite of overall similarity of secondary structure (and predicted tertiary structure), the yeast and animal PPR motifs differ from their plant counterparts, and are generally more divergent in sequence (Lipinski et al. 2011; Rackham and Filipovska 2012; Herbert et al. 2013). It is thus not clear to what extent the mechanisms of RNA substrate recognition elucidated for plant PPRs can be applied to non-plant members of the family. Mechanisms of RNA substrate recognition or exact sequences of recognized sites have not been identified for any of the fungal or animal PPR proteins.

The genome of *Saccharomyces cerevisiae* encodes 14 typical PPR proteins, all involved in the expression of mitochondrial genes (Lipinski et al. 2011; Herbert et al. 2013). They function as RNA stability and/or translation factors, both general, and gene-specific (for review, see Herbert et al. 2013). Additionally, the yeast mitochondrial RNA polymerase (Rpo41p) contains five divergent PPR motifs, that are not essential for its function (Kruszewski and Golik 2016).

One of the most interesting aspects of PPR protein biology is related to their role in evolution. Nucleo-mitochondrial incompatibility was found to play a role in speciation, as a variant of the Dobzhansky–Muller reproductive barrier (Lee et al. 2008; Chou and Leu 2010; Levin et al. 2014; Spírek et al. 2015). In yeasts, the interactions between the rapidly evolving mitochondrial sequences and the nuclear-encoded PPR proteins contribute to such incompatibility in the case of Aep2p interacting with the *ATP9* mRNA (Lee et al. 2008), and Dmr1p (Ccm1p) interacting with the 15S rRNA (Jhuang et al. 2017). Other yeast PPR proteins are also likely to be involved in speciation through nucleo-mitochondrial incompatibility, although detailed evidence is not yet available (Jhuang et al. 2017). Consequently, genes encoding PPR proteins undergo rapid evolution and are among the most divergent in pairwise ortholog comparisons between yeast species (Lipinski et al. 2011).

Prior studies, using genetic, molecular, and evolutionary approaches, indicated that the primary target of the PPR protein encoded by the yeast *DMR1* (*YGR150C,* also known as *CCM1*) gene is the mitochondrial small subunit rRNA (15S rRNA) (Puchta et al. 2010). Deletion of the *DMR1* gene leads to degradation of 15S rRNA (Puchta et al. 2010), followed by the loss of mtDNA stability and a complete respiratory deficiency, and hypomorphic point mutations lead to a decrease in the 15S rRNA level and reduced mitochondrial translation (Lipinski et al. 2011). Replacing the *S. cerevisiae DMR1* gene with its ortholog from *S. bayanus* also results in a decrease of mature 15S rRNA level, with concomitant defects in translation, leading to a marked (but partial) respiratory deficiency (Jhuang et al. 2017). Parallel studies suggested that this protein could be involved in the splicing of the fourth intron in the *COB* and *COX1* transcripts (hence the name *CCM1*) (Moreno et al. 2009, 2012). Subsequent studies indicate, however, that this is likely to be a secondary effect, caused by the loss of maturase protein expression following the loss of translation in the mutant (Puchta et al. 2010). The observations that neither *COB*, nor *COX1* mRNA expression and processing are affected in *S. cerevisiae* strains where the native Dmr1 protein is replaced by the *S. bayanus* ortholog, and that the nucleo-mitochondrial incompatibility in these strains seems to be limited to the expression of 15S rRNA (Jhuang et al. 2017), further confirm the identification of 15S rRNA as the sole primary RNA target of the Dmr1 protein.

In this study we performed detailed in vitro studies of the interaction between Dmr1p and 15S rRNA, identifying a distinct motif of at least 14 nucleotides, composed of repeats of the AUA triplet, occurring in two regions of the 15S rRNA sequence, as the proposed recognition site.

## RESULTS

### Identification of the fragments of the 15S rRNA molecule recognized by Dmr1p

Previous work indicated that Dmr1p binds the 15S rRNA, consistent with its role in ensuring this molecule's stability inferred from genetic studies (Puchta et al. 2010). This binding is not, however, limited to a single target within 15S rRNA sequence, as at least three large regions were bound. In order to determine the precise localization of the RNA sequences recognized by Dmr1p, we performed an in vitro cross-linking assay using recombinant Dmr1p and fragmented 15S rRNA.

The Dmr1-MBP-His_6_ protein (Puchta et al. 2010) was expressed in *E. coli* and purified on a HisTrap HP Ni-NTA column and subsequently on HiLoad Superdex 200 pg size exclusion column. Radiolabeled full-length 15S rRNA sequence obtained by in vitro transcription was fragmented using magnesium cations at high temperature and incubated with the Dmr1-MBP-His_6_ protein. Cross-linked RNA–protein complexes were then bound to HisPur cobalt resin. The supernatant with the unbound RNA fragments was removed, the resin was washed three times, and the supernatants from each wash were pooled together with the original supernatant as the unbound RNA fraction. Protein with bound RNA fragments was then eluted from the resin, and RNA was released by proteinase K/SDS treatment, followed by phenol/chloroform extraction, resulting in the bound RNA fraction.

**FIGURE 1.**
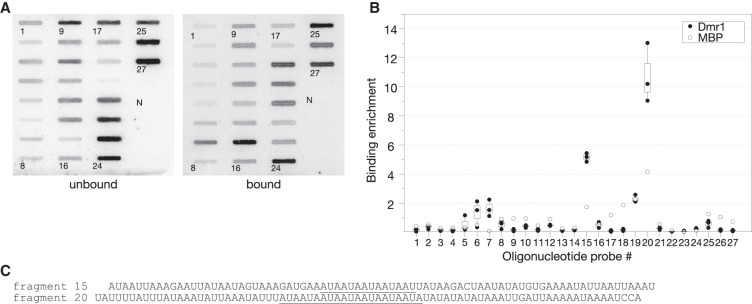
Identification of the fragments of radiolabeled 15S rRNA coprecipitating with Dmr1p. (*A*) Slot-blot macro array hybridization of the RNA fragments from the unbound and bound fraction with 27 80-nt oligonucleotide probes (numbers to the *left* of respective slots, probe #1 corresponds to the 3′ end of the molecule, and probe #27 to the 5′ end) covering the entire length of the 15S rRNA sequence. N is an unrelated negative control probe. A representative result of three replicates is shown. Details of the procedure are described in the text. (*B*) Ratios of bound to unbound signal for each probe. Values from three replicates with Dmr1p-MBP-His_6_ fusion protein are shown as black circles. Open circles represent results obtained using the MBP-His_6_ tag as negative control. (*C*) Sequences of 15S rRNA fragments complementary to probe #15 and probe #20 showing the highest bound to unbound ratio. AUA trinucleotide repeats unique to these two fragments are underlined.

The bound and unbound RNA preparations were subsequently hybridized with a slot-blot array of 27 complementary 80 nt oligonucleotides covering the entire 15S rRNA sequence (overlapping by 20 nt). Hybridization signal from each probe was quantified and the ratio of bound/unbound RNA complementary to each of the nucleotides calculated (Fig. 1A,B). The results indicate that for the majority of probes there is a very low background enrichment of corresponding RNA fragments in the bound fraction, comparable to the negative control (a sequence unrelated to yeast mtDNA). Only in two regions, with peaks around probe #15 and probe #20 we observed a very strong increase in the hybridization signal in the bound fraction relative to the unbound supernatants (Fig. 1B). A third, weaker peak was observed around probe #7.

Analysis of RNA sequences complementary to the probes #15 and 20#, that exhibited strong binding to Dmr1p in vitro, revealed that they share identical repetitive stretches comprising several repeats of the AUA trinucleotide (Fig. 1C). The fragment recognized by probe #15 contains the 14-nt (AUA)_4_AU motif, whereas the fragment recognized by probe #20 contains a longer 21-nt stretch of (AUA)_7_. Even though the entire 15S rRNA sequence is AU-rich, there are no other occurrences of more than four AUA repeats in other regions of the molecule. The fragment complementary to probe #7, that also showed some weak binding, does not contain an uninterrupted (AUA)_n_ tract. There is, however, a somewhat similar sequence (AUA)_2_UGGGUAAUA, and this fragment was included in further experiments.

The (AUA)_4_AU motif occurs only twice in the mature 15S rRNA sequence, at positions 457 [the longer (AUA)_7_ stretch, #20], and 761 (#15). Additionally, it can be found in the 5′ extension of the 15S rRNA primary transcript which is removed by processing (Osinga et al. 1981; Puchta et al. 2010), at position −58 (relative to the first nucleotide of the mature rRNA). An in silico search of the entire yeast mtDNA sequence reveals multiple additional occurrences of this motif. Many are located in noncoding untranscribed regions, but there are multiple copies in the sequences of primary transcription units (identified after Turk et al. 2013). With the exception of the *VAR1* ORF, they are found outside the coding or functional RNA sequences, often around the tRNA genes contained in longer polycistronic transcript. It is unclear whether their presence there has any functional relevance (see Discussion).

Copurification of bacterial RNA-binding factors, most notably Hfq, with heterologous proteins containing the hexahistidine tag and purified by metal-affinity chromatography is a known source of false positive RNA binding activities (Milojevic et al. 2013). As an additional control we have therefore performed a similar procedure using a vector expressing only the MBP-His_6_ tag. The MBP-His_6_ protein was cross-linked to fragmented radiolabeled 15S rRNA, and the ratio of bound/unbound RNA fragments was calculated using the same method as described above for the Dmr1-MBP-His_6_ protein. Some enrichment of the AU-rich fragments was observed, but the bound/unbound ratio was always much lower than for the Dmr1-MBP-His_6_ protein (Fig. 1B, open circles), proving that the result observed for probes #15 and #20 was due to specific interactions between Dmr1p and the RNA fragments. Nevertheless, for subsequent experiments we introduced an additional purification step on a size exclusion column. The preparation obtained following size exclusion was also analyzed by mass spectrometry (LC–MS–MS/MS), and no contamination with Hfq or other known bacterial RNA-binding proteins was detected (identified peptides are listed in Supplemental Table S2).

In order to confirm that the identified AUA trinucleotide repeats constitute the specific Dmr1p binding site, we performed a series of electrophoretic mobility shift assays (EMSA). For these experiments the MBP-His_6_ tag was removed from the heterologously expressed Dmr1-MBP-His_6_ protein using TEV protease, followed by size-exclusion chromatography to obtain highly purified Dmr1p. Radiolabeled RNA substrates were obtained by T7 in vitro transcription on synthetic oligonucleotide templates, and purified by polyacrylamide gel electrophoresis.

**FIGURE 2.**
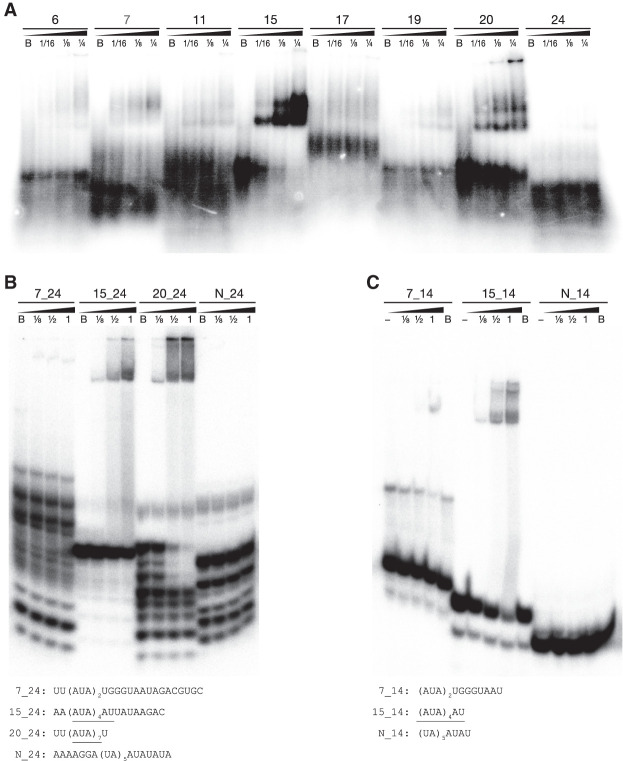
Gel shift (EMSA) assays show specific binding of Dmr1p to RNA fragments containing the trinucleotide AUA repeats. (*A*) Radiolabeled 80 nt RNA fragments corresponding to probes #15 and #20 clearly show a mobility shift indicative of binding by increasing concentrations of Dmr1p. Fragment #7 and other fragments bind Dmr1p weakly or not at all. (*B*) Radiolabeled 24 nt fragments containing the 14- or 21-nt stretch of AUA repeats (fragments 15_24 and 20_24) show mobility shift indicative of binding by Dmr1p, whereas the fragment containing fewer repeats (7_24) and a control fragment containing dinucleotide UA/AU repeats (N_24) do not interact with the protein. (*C*) Radiolabeled 14 nt fragment comprising the minimal recognized motif (AUA)_4_AU (15_14) shows clear mobility shift by increasing amounts of Dmr1p, whereas the fragment containing fewer repeats (7_14) exhibits only minimal shift at the maximum Dmr1p concentration, and the control fragment containing dinucleotide UA/AU repeats (N_14) shows no interaction. Dmr1p concentrations increase from *left* to *right* for each fragment, and are labeled by fractions of the maximum (1), which corresponded to 0.8 µg per reaction. Total protein amount in each reaction was kept constant at 0.8 µg by addition of BSA. B is negative control (only BSA), “-” denotes negative control with no protein. Sequences of fragments used in *B* and *C* are shown *below* respective gels, with the AUA trinucleotide repeats underlined. The autoradiograms (originally recorded as high bit depth TIFF files) were linearly transformed to visualize weaker bands, resulting in an overexposure of the stronger bands.

The first series of experiments, using long RNA substrates corresponding to the probes used in the coprecipitation experiment, confirmed the interaction of Dmr1p with fragments complementary to probes #15 and #20 and a very weak interaction with the fragment complementary to probe #7. The fragments corresponding to probes #6, #11, #17, #19, and #24 showed very weak or no appreciable interaction with Dmr1p (Fig. 2A). At the highest Dmr1 protein concentration, a second super-shifted band appears for probes #15 and #20, suggesting possible dimerization of the bound protein, or formation of higher-order complexes through RNA–RNA interactions, facilitated by the repetitive nature of the sequences.

As these preliminary results indicated visible background binding to AU-rich sequences in addition to clear interaction with the regions identified in the coprecipitation experiment, we designed shorter (24 nt and 14 nt) RNA substrates comprising the putative Dmr1-binding motif of fragment #15 and #20, as well as the weakly bound fragment #7. Unrelated, but similarly AU-rich fragments were used as negative controls. The results obtained with longer, 24 nt RNA transcripts (Fig. 2B) clearly indicate that the long (AUA)_7_ stretch (found in fragment #20) strongly binds Dmr1p in vitro, and the shorter (AUA)_4_AU motif is still sufficient to ensure reliable binding. The substrate containing the (AUA)_2_UGGGUAAUA sequence found in fragment #7 showed only minimal binding, and the negative control did not exhibit any mobility shift indicative of protein binding. In the assay using shorter, 14 nt substrates, the (AUA)_4_AU sequence (common for fragments #15 and #20) showed clear binding (Fig. 2C), while the (AUA)_2_UGGGUAAUA sequence showed only marginal binding at the highest protein concentration, and the negative control with the distinct, but similarly repetitive AU-rich sequence (UA)_5_AUAU was not bound at all. In the experiment using 24 nt probes, and to a lesser extent also the 14 nt probes, even unbound RNAs migrate in the nondenaturing gel as multiple bands, indicating that they easily form different secondary structures which may be relevant for their interactions with Dmr1p.

The EMSA assay results have thus confirmed that the sequence containing four AUA repeats (plus an additional AU) is specifically recognized by the Dmr1 protein, and that the addition of more repeats (to seven) seems to increase the strength of interaction. Binding to a sequence containing fewer AUA repeats cannot be excluded, but is markedly weaker, whereas other AU-rich sequences do not interact with Dmr1p sufficiently to cause a detectable electrophoretic mobility shift.

### Dmr1p binding protects RNA oligonucleotides from exoribonuclease degradation in vitro

As many of the known PPR proteins stabilize their target RNAs (Andres et al. 2007; Delannoy et al. 2007; Schmitz-Linneweber and Sluyter 2008; Pfalz et al. 2009; Johnson et al. 2010; Kühl et al. 2011; Prikryl et al. 2011; Herbert et al. 2013) and deletion of the *DMR1* gene results in fragmentation of the 15S rRNA (Puchta et al. 2010) in vivo, we decided to verify if the binding of Dmr1 protein can protect RNA from the activity of ribonucleases in vitro.

**FIGURE 3.**
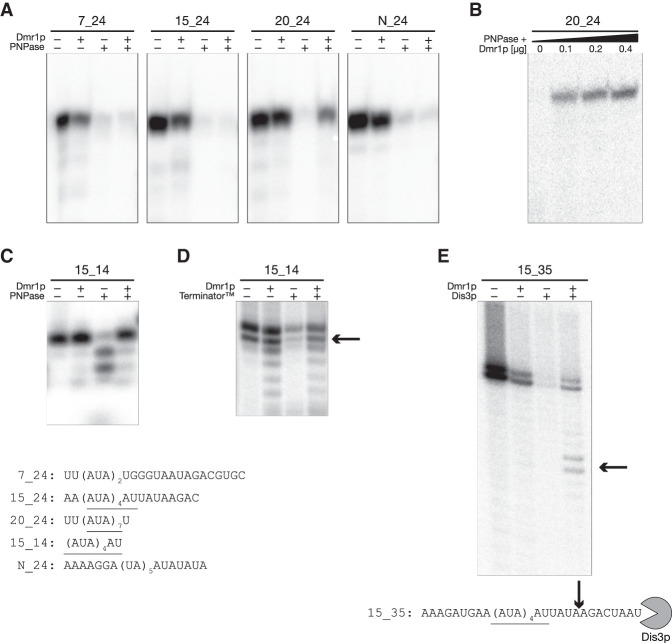
Binding by Dmr1p protects specifically recognized RNA fragments from exoribonuclease degradation. (*A*) Binding by Dmr1p protects the 24 nt fragment containing seven AUA repeats (20_24) from degradation by 3′–5′ exoribonuclease—polynucleotide phosphorylase. An amount of 1.2 µg of Dmr1-His_6_ fusion protein was preincubated with the radiolabeled RNA substrate prior to the addition of recombinant *Synechocystis sp.* PNPase. (*B*) Increasing the amount of Dmr1p increases the fraction of radiolabeled RNA (20_24) protected from degradation by PNPase. Total amount of recombinant Dmr1-His_6_ fusion protein is indicated for each lane. (*C*,*D*) Binding by Dmr1p protects the 14 nt radiolabeled RNA corresponding to the (AUA)_4_AU recognition motif (15_14) from degradation by 3′–5′ exoribonuclease—PNPase (*C*) and 5′–3′ exoribonuclease—Terminator (*D*). The radiolabeled substrate was treated with 5′ polyphosphatase prior to the assay. The monophosphorylated substrate that is susceptible to the 5′–3′ exoribonucleolytic activity of Terminator is indicated with an arrow. An amount of 1.2 µg of Dmr1-His_6_ fusion protein was preincubated with the radiolabeled RNA substrate prior to the addition of the nucleases as described in Materials and Methods. (*E*) Dmr1p bound to the 35 nt substrate stops degradation by recombinant Dis3 3′–5′ exoribonuclease at a site 4–5 nt downstream from the (AUA)_4_AU recognition motif (marked by arrow). Sequences of fragments used in each assay are shown *below* respective gels, with the AUA trinucleotide repeats underlined.

Heterologously expressed Dmr1-His_6_ protein purified by metal-affinity chromatography followed by size-exclusion was used in these experiments. Radiolabeled RNA substrates were obtained by T7 in vitro transcription on synthetic oligonucleotide templates, and purified by polyacrylamide gel electrophoresis. First, the 24 nt RNA fragments, same as those used in the EMSA experiments described above were incubated with Dmr1p and the commercially available recombinant 3′–5′ exoribonuclease—polynucleotide phosphorylase (PNPase). The substrate containing the longest (AUA)_7_ binding site (UU(AUA)_7_U) was completely protected from PNPase degradation by bound Dmr1p, whereas fragments containing the core 14 nt (AUA)_4_AU sequence, and the possibly weakly interacting (AUA)_2_UGGGUAAUA, as well as the negative control with the distinct, but similarly AU-rich sequence were not protected and efficiently degraded by PNPase (Fig. 3A). Incubation of the UU(AUA)_7_U substrate with different amounts of recombinant Dmr1p (Fig. 3B) demonstrated that while protection was observed even at the lowest concentration tested (0.1 µg protein per reaction), the amount of undegraded RNA visibly increased when more protein was added.

The lack of protection of the 24 nt fragment containing the core 14 nt (AUA)_4_AU sequence could be a result of weaker Dmr1p binding, but it could also be attributed to the fact that this substrate contains eight additional nucleotides downstream from the putative Dmr1p binding sequence (Fig. 3A), thus enabling the 3′–5′ processive exoribonucleolytic activity of PNPase to commence degradation and displace the bound protein. The protected RNA fragment contained only one nucleotide downstream from the (AUA)_7_ repeats (Fig. 3A). We have therefore tested whether a short 14 nt RNA substrate, containing only the core (AUA)_4_AU sequence could be protected by Dmr1p from degradation by PNPase. The results (Fig. 3C) indicate that at least partial protection can be achieved in these conditions.

We have also tested whether binding of Dmr1p to the 24 nt fragment containing the core 14nt (AUA)_4_AU sequence can protect it from degradation from the 5′ end, where only two additional nucleotides can be found upstream of the putative binding site. The transcript was treated with 5′ polyphosphatase to generate an RNA fragment monophosphorylated at the 5′ end. Incubation of this substrate with a commercially available 5′–3′ exoribonuclease (Terminator) indicates that partial but evident protection can be achieved (Fig. 3D).

Even though some unspecific background RNase activity (difficult to avoid when RNA substrates are incubated for a prolonged time with protein preparations in a buffer optimal for RNA degradation) in the Dmr1 protein preparation is apparent, both experiments confirm that binding of Dmr1 to the recognized motif is specific and strong enough to protect from exoribonucleolytic activity from either end.

Protection of RNA substrates from exoribonucleolytic degradation was used to further confirm the identified core binding site in a footprinting assay (Fig. 3E). A 35 nt radiolabeled RNA substrate containing the minimal (AUA)_4_AU binding sequence with additional 12 nt downstream and 9 nt upstream was incubated with Dmr1p and a recombinant yeast processive hydrolytic 3′–5′ exoribonuclease Dis3p (Rrp44p) (Dziembowski et al. 2007). In the absence of Dmr1p the substrate was efficiently (albeit not completely) degraded by Dis3p, and the presence of Dmr1p provided protection. A partial degradation product was observed in the presence of Dmr1p, corresponding to the substrate with 8–9 nt removed from the 3′ end, suggesting that degradation stopped 3–4 nt downstream from the putative (AUA)_4_AU Dmr1p binding site. This is consistent with the fact that Dis3p leaves a final 3–5 nt undegraded product (Dziembowski et al. 2007; Lorentzen et al. 2008), and further confirms our prediction that the (AUA)_4_AU motif is the site recognized and bound by Dmr1p.

### Coevolution of Dmr1p and its target sequence in *Saccharomycetales*

**FIGURE 4.**
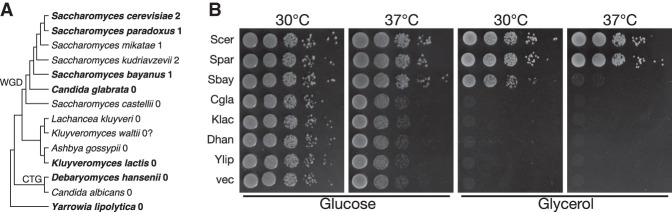
Compatibility of yeast Dmr1p orthologs with *S. cerevisiae* mtDNA correlates with the presence of the (AUA)_4_AU recognition motifs in the 15S rRNA sequence. (*A*) Cladogram representing the phylogeny of 14 selected representatives of *Saccharomycetales* based on the published fungal maximum likelihood tree (Fitzpatrick et al. 2006). *DMR1* orthologs from species in bold type were tested for compatibility in this work (*S. mikatae* and *S. kudriavzevii* were also found compatible in Jhuang et al. 2017). Numbers denote the occurrences of the (AUA)_4_AU motif in 15S rRNA sequences of each species. (WGD) Whole genome duplication, (CTG) the clade with nonstandard nuclear genetic code (CTG encodes serine). The sequence from *K. waltii* was not complete, which is denoted by the question mark. (*B*) Respiratory competence of Δ*dmr S. cerevisiae* strains expressing *DMR1* orthologs from *S. paradoxus* (Spar), *S. bayanus* (Sbay), *C. glabrata* (Cgla)*, Kluyveromyces lactis* (Klac)*, Debaryomyces hansenii* (Dhan), and *Yarrowia lipolytica* (Ylip). The *S. cerevisiae* (Scer) *DMR1* gene was used as a positive control, and the empty vector was used as a negative control (vec). Overnight cultures in complete synthetic medium (CSM) without leucine were spotted in a series of 10-fold dilutions, on fermentable (glucose) and respiratory (glycerol) media and incubated at 30°C (normal) and 37°C (restrictive) for 3 d.

PPR proteins evolve rapidly (Lipinski et al. 2011), and their interaction with mitochondrial transcripts is often the basis of nucleo-mitochondrial incompatibility playing an important role in speciation (Lee et al. 2008; Chou et al. 2010; Chou and Leu 2010). Interaction of Dmr1p with 15S rRNA was recently found to be involved in hybrid incompatibility between *S. cerevisiae* and *S. bayanus*, which likely arose upon the divergence between *S. bayanus* and the common ancestor of *S. cerevisiae, S. paradoxus, S. mikatae*, and *S. kudriavzevii* (Jhuang et al. 2017). In order to assess the role of the identified Dmr1p binding site in the evolution of nucleo-mitochondrial compatibility, we searched for the identified (AUA)_4_AU Dmr1p binding site in 15S rRNA sequences of 14 representative members of *Saccharomycetales* (Fig. 4A). In the *Saccharomyces sensu stricto* clade two occurrences of this motif can be found in *S. cerevisiae* and in *S. kudriavzevii*. In *S. paradoxus, S. mikatae*, and *S. bayanus*, however, only one motif (in the region corresponding to fragment #15) is preserved, whereas in *S. castellii, Candida glabrata,* and all the species that diverged prior to the Whole Genome Duplication (WGD) this motif cannot be found anywhere in the 15S rRNA sequence. Remarkably, Dmr1p from *S. mikatae* and *S. kudriavzevii,* as well as from *S. paradoxus,* can function in the context of *S. cerevisiae* mtDNA (Jhuang et al. 2017). The ability of *S. bayanus* Dmr1p to support the stability of *S. cerevisiae* 15S rRNA is reduced, evidenced by significantly slower, but not entirely abolished respiratory growth (Fig. 4B; Jhuang et al. 2017), as well as reduced level of the mature rRNA and weaker protein–RNA interaction (Jhuang et al. 2017).

In order to provide experimental confirmation of these findings, we replaced the *S. cerevisiae DMR1* gene with orthologs from *S. paradoxus*, *S. bayanus*, *C. glabrata, Kluyveromyces lactis, Debaryomyces hansenii*, and *Yarrowia lipolytica* using the plasmid shuffling strategy, and tested the respiratory competence of the obtained strains by assaying growth on glycerol at normal and elevated temperature (Fig. 4B). The ortholog from *S. paradoxus,* the closest tested relative of *S. cerevisiae*, functioned as well as the native gene, supporting respiratory growth in both conditions, similar to previous reports (Jhuang et al. 2017). Partial incompatibility between *S. bayanus DMR1* and *S. cerevisiae* mtDNA manifested itself by markedly slower respiratory growth at normal temperature, and a complete respiratory deficiency at the elevated temperature. The respiratory-deficient transformants also display slower growth on glucose, typically observed in *petite* yeast mutants. A similar phenotype was reported recently by another group (Jhuang et al. 2017). *DMR1* orthologs from the remaining more distant relatives that do not have any (AUA)_4_AU motifs in the 15S rRNA sequence failed to support any perceptible respiratory growth, signifying a complete lack of compatibility with *S. cerevisiae* mtDNA.

To further strengthen these results, we performed a reverse in silico analysis, searching for sequence motifs that are at least 14 nt long, occur in the *S. cerevisiae* 15S rRNA sequence in at least two distinct regions, are conserved in the *Saccharomyces* species that show at least partial compatibility of Dmr1p orthologs with *S. cerevisiae*, and are absent from the species where the Dmr1p ortholog is completely incompatible with *S. cerevisiae* mtDNA. The regions containing the (AUA)_4_AU motif are the only ones that fulfil these criteria (code and sequences used in this analysis are available at github.com/golikp/dmr1_motif).

Overall, Dmr1p orthologs from species that do not have any occurrences of the identified (AUA)_4_AU motifs in their 15S rRNA completely fail to function in the context of *S. cerevisiae* mtDNA, while those originating from species where at least one such motif can be found will at least partially complement the phenotype of a *S. cerevisiae* Δ*dmr1* strain (Fig. 4B; Jhuang et al. 2017). This further confirms the identification of the Dmr1p binding site, and provides a clear example of the involvement of PPR proteins in evolving hybrid incompatibility in yeasts. There is, however, no clear correlation between the number of these motifs and the degree of functional conservation, as *S. paradoxus* Dmr1p (one motif) is more compatible with *S. cerevisiae* 15S rRNA than *S. bayanus* Dmr1p (also one motif), suggesting that other factors can influence the strength of Dmr1p–15S rRNA interaction.

### Structure model of Dmr1p indicates that yeast PPR motifs are highly divergent and may not be recognized by consensus-based methods

**FIGURE 5.**
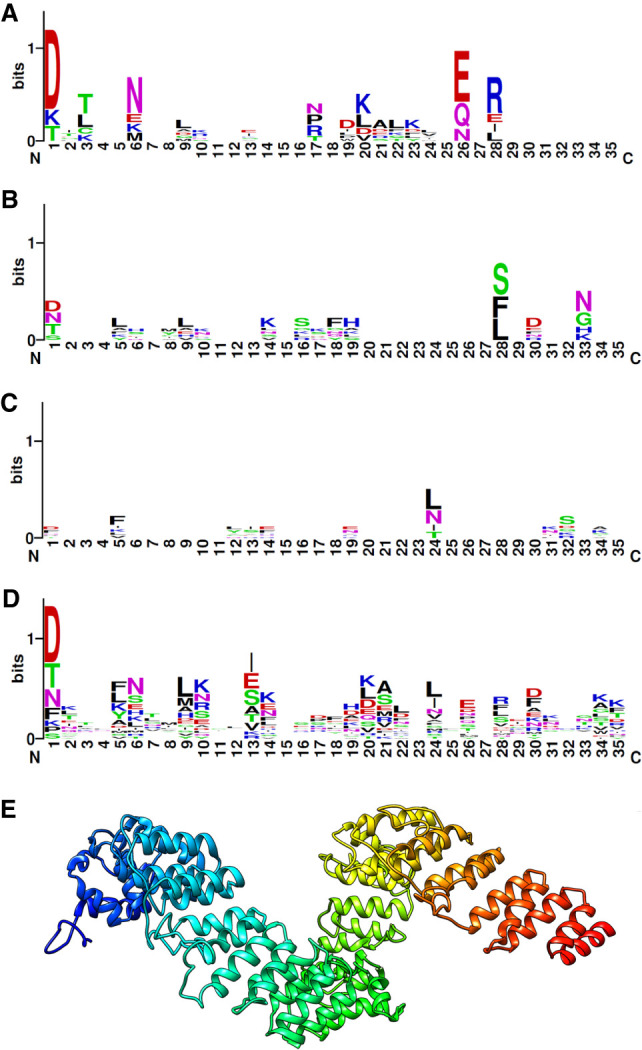
Sequence conservation of PPR motifs in Dmr1p. Sequence logos representing amino acid conservation were generated using WebLogo (Crooks et al. 2004) for every third PPR motif in Dmr1p sequence starting from (*A*) the first, (*B*) the second, (*C*) the third motif. (*D*) A summary logo for all the PPR motifs in the Dmr1p sequence. (*E*) The predicted structure model of Dmr1p (C-score = −0.76, estimated RMSD = 11.1 ± 4.6Å) obtained by homology modeling.

Yeast and animal PPR motifs are more divergent than the ones found in plants (Herbert et al. 2013), and their identification in silico is less accurate (Lipinski et al. 2011). In order to improve the identification of PPR motifs, we obtained a model of the Dmr1p structure (Fig. 5E) using the I-TASSER server (Yang and Zhang 2015). The highest scoring model (C-score = −0.76) contained 21 predicted pairs of antiparallel α-helices that are the hallmark of PPR repeats, which span the entire length of the protein. Other, lower-scoring models differed in the tertiary structure, but predicted similar secondary structure with the same number of α-helices. This corresponds well to the length of the longer 21 nt target sequence within 15S rRNA. It is also an indication, that current sequence-based methods for identifying PPR motifs are still inadequate as far as the PPR proteins in Opisthokonta are concerned.

Studies correlating the sequences of individual PPR motifs with their target RNA sequences in plants revealed the existence of a simple combinatorial code wherein two amino acids in two adjacent α-helical segments of a PPR motif determine the identity of a single nucleotide in the recognized sequence (Barkan et al. 2012; Yagi et al. 2013; Gully et al. 2015; Hall 2016; Harrison et al. 2016; Yan et al. 2019). Attempting to apply these principles to the predicted PPR motifs of Dmr1p failed to yield significant results, suggesting that the target recognition mechanisms in non-plant PPR proteins may be different. All of the conclusions in this section are based solely on the in silico predictions, due the absence of experimentally verified structures for non-plant PPR proteins.

## DISCUSSION

Earlier studies in yeast and animal cells identified transcripts bound by PPR proteins, in certain cases narrowing the interaction down to a particular region. A 64 nt sequence in the 5′UTR of yeast *COB* mRNA was implicated in the interaction with the Cbp1 protein through suppressor analysis (Chen and Dieckmann 1997), and a short 38 nt region of the *COX2* 5′UTR, containing a stem–loop structure, was identified as the target of Pet111p (Dunstan et al. 1997), but available data were not sufficient to establish whether a linear sequence motif was responsible for these interactions. Only a recent study revealed, that Pett111p binds two RNA sequences within the 5′UTR and at the beginning of *COX2* ORF (Jones et al. 2019). However, both identified sequences share little evident similarity, making any conjectures as to the recognition mechanism problematic. Unlike in plants, prior studies of yeast (as well as animal) PPR proteins failed to identify the exact target sequence or a predictable recognition code. The only non-plant PPR proteins with clearly determined target sequences—KRIPP11 and KPAF4 from the protist *Trypanosoma brucei,* recognize simple polynucleotide repeats of poly(G) and poly(A), respectively (Kamba et al. 2018; Mesitov et al. 2019).

The 15S rRNA was previously identified as the primary, and likely sole target of yeast Dmr1p through a combination of genetic, molecular and evolutionary studies (Puchta et al. 2010). By coprecipitation of a heterologously expressed Dmr1p with fragmented radioactive 15S rRNA we identified two regions that preferentially bind to the protein. Both contain repetitive stretches comprising several repeats of the AUA trinucleotide, either as a shorter 14-nt (AUA)_4_AU motif, or a longer 21-nt stretch of (AUA)_7_. Both regions correspond to regions near the surface of mitoribosome (Desai et al. 2017). Preferential binding of Dmr1p to these sequences was further confirmed in gel-shift (EMSA) and RNase protection experiments. Other similarly AU-rich fragments with a different sequence showed much weaker interaction in all these assays, suggesting that the interaction of Dmr1p with its RNA substrate is, at least partially, sequence-specific. In silico survey of the 15S rRNA sequence indicates that it contains numerous instances of shorter AUA repeats (one UA(AUA)_3_, one A(AUA)_2_A, five (AUA)_2_A, and eight (AUA)_2_), yet fragments containing them failed to yield significant enrichment in the RNA pull-down experiment, suggesting that the 14-nt (AUA)_4_AU motif is close to being the minimal target sequence required for Dmr1p binding.

Additional evidence supporting the identification of AUA repeats as the preferred target recognized by Dmr1p comes from in vitro RNase protection assays. Only the RNA fragments containing the minimal (AUA)_4_AU motif or the longer (AUA)_7_ sequence were protected from exoribonucleolytic activity by incubation with Dmr1p. The shorter (AUA)_4_AU motif did not provide protection from degradation by the 3′–5′ exoribonuclease PNPase when the substrate contained eight additional nucleotides downstream, suggesting that the strong processive activity of PNPase is sufficient to displace bound Dmr1p from this RNA. Binding of Dmr1p to a similar substrate was, however, sufficient to arrest the degradation by another 3′–5′ exoribonuclease—Dss1p, at a site consistent with the occupation of the (AUA)_4_AU motif by the bound Dmr1p. Protecting the 15S rRNA from degradation by ribonucleases was proposed to be the primary function of Dmr1 protein, based on the distinct degradation pattern observed in the deletant (Puchta et al. 2010). Caution is, however, required in interpreting the results of our in vitro protection assays in the context of the presumed in vivo function of Dmr1p. The ribonucleases used in these assays are not, with the possible exception of Dis3p (Turk et al. 2013), normally found in yeast mitochondria, and the results obtained using PNPase and the longer substrate with the core (AUA)_4_AU motif suggest that the interaction of 15S rRNA with Dmr1p alone may not be enough to protect from the mtEXO complex, which contains an RNA helicase activity (Dziembowski et al. 2003; Malecki et al. 2007; Szczesny et al. 2012). The protective effect of Dmr1p binding could be enhanced by the features of rRNA structure and/or interactions with other proteins that were not re-created in the simple in vitro system.

Whereas the in vitro RNase protection assays performed in this study were sufficient to confirm that Dmr1p binds to the identified sequence motif, they cannot be directly used to explain its function in vivo. Deletion of *DMR1* results in the fragmentation of 15S rRNA (Puchta et al. 2010), but the pattern of this fragmentation is not obviously related to the location of the motif identified in the present study. Partial loss of *DMR1* function results in 15S rRNA depletion, but without an apparent fragmentation pattern (Lipinski et al. 2011; Jhuang et al. 2017). It is therefore not clear, how binding of Dmr1 to 15S rRNA protects it from degradation. In the absence of more detailed in vivo studies any hypotheses concerning the mechanism of its action remain purely speculative. Such in vivo experiments are challenging, as the absence of Dmr1p leads to massive and pleiotropic dysfunction of the mitochondrial genetic system, related to the loss of mtDNA stability (conversion to *rho*^−^/*rho*^*0*^ cytoplasmic petites) with concomitant disturbance of translation and RNA processing. Based on the observed presence of Dmr1p within the large ribosome-bound complex (Kehrein et al. 2015), its critical role in the ribosome assembly, and the general function of PPR proteins as adaptors in RNA–protein interactions (Schmitz-Linneweber and Sluyter 2008), it is tempting to postulate that its role is related to assisting the assembly of the nascent 15S rRNA transcript and ribosomal proteins into the functional subunit.

In plants, PPR proteins recognize their target sequences by a deterministic modular code, where one motif (i.e., one pair of α-helices) corresponds to a single nucleotide in the RNA (Barkan et al. 2012; Ban et al. 2013; Ke et al. 2013; Takenaka et al. 2013; Yagi et al. 2013; Yin et al. 2013). As the key amino acids recognizing a nucleotide belong to two adjacent repeats, a protein containing N PPR motifs is expected to recognize a sequence N − 1 nucleotides long (Herbert et al. 2013). Yeast (and animal) PPR motifs differ from the plant consensus in sequence (Rackham and Filipovska 2012; Herbert et al. 2013), and are generally more divergent (Lipinski et al. 2011; Herbert et al. 2013), but are nevertheless broadly similar in size, and comparative modeling suggests that they fold into a similar superhelical shape. It is thus reasonable to presume that the relationship of the number of helical motifs to the length of the bound RNA sequence should be similar in non-plant and plant PPR proteins.

As the sequence recognized by Dmr1 consists of trinucleotide AUA repeats, we turned to structural predictions obtained via the I-TASSER server, looking for regularities in the motif sequences. We compared sequence profiles in every third motif in three possible phases (Fig. 5). Two of these comparisons (Fig. 5B,C) did not yield any significant results, but in one phase (starting with the first identified motif) we noticed a conservation of aspartate and asparagine, at position 1 and 6, respectively (Fig. 5A). In plants, the presence of asparagine in position 6 indicates a preference for binding pyrimidine (uridine or cytosine) in every combination, while aspartate in position 1 facilitates binding to keto group nucleotides (uridine or guanine) by the preceding motif (Barkan et al. 2012; Yagi et al. 2013; Gully et al. 2015). It is tempting to relate finding these amino acid residues in every third PPR motif of Dmr1p to the occurrence of uridine at every third nucleotide of the recognized RNA sequence, but the limited data available make such conjecture purely speculative. Additionally, it is not evident from these results if either the position 1 or 6 plays the leading role in sequence specificity. One must also consider the possibility, that as is the case with PUF proteins, the malleability of both protein and RNA structure may lead to deviations from the one motif, one nucleotide model (Hall 2016). Lack of obvious conservation in the remaining PPR motifs suggests that the similarity to the plant PPR substrate recognition mechanism is, at best, limited (assuming the validity of our I-TASSER structural predictions).

Comparative modeling of the Dmr1 protein structure suggests the presence of 21 pairs of antiparallel α-helices. The shorter identified recognized motif is 14 nt long, which is a number close to the 15 core motifs containing the repetitive D_1_ N_6_ pattern. However, the longer of the recognized motifs reaches the length of exactly 21 nt. The general rule of one PPR motif recognizing a single RNA nucleotide appears thus to be valid for the interaction of Dmr1p with its target sequence, yet, not all of the predicted pairs of α-helices conform to the current HMM profiles of yeast PPR motifs (Lipinski et al. 2011), and their participation in substrate recognition is only hypothetical. Further experimental verification, involving Dmr1p with substitution of amino acids in these crucial positions is required. Experiments conducted on plant PPR scaffolds indicate, that if our predictions regarding the similarities in recognition mechanisms are correct, obtaining a synthetic Dmr1p protein, that binds either a poly(A) or poly(U) sequence should be possible (Coquille et al. 2014)

One of the most fascinating aspects of PPR protein biology is their role in the evolution of nucleo-organellar compatibility. In plants, one of their postulated roles is maintaining the function of organellar genes by compensating for mutational pressure, known as “genome debugging” (Maier et al. 2008; Tillich et al. 2010). In yeasts they maintain and regulate the expression of mitochondrial genes, and undergo very rapid evolution to keep up with the highly divergent organellar transcripts (Lipinski et al. 2011; Herbert et al. 2013). Compatibility between orthologous PPR proteins and their target RNAs is quickly lost even in closely related yeast species, contributing to the emergence of reproductive isolation (Lee et al. 2008; Chou and Leu 2010). Compatibility with *S. cerevisiae* mtDNA is retained for Dmr1p orthologs from *S. paradoxus, S. mikatae*, and *S. kudriavzevii*, whereas the Dmr1 protein from the more distant *S. bayanus* is only partially compatible, as its interaction with *S. cerevisiae* 15S rRNA is weaker, leading to a slower respiratory growth at normal temperature, and a complete respiratory deficiency at restrictive (elevated) temperature, with concomitant decrease in the mature 15S rRNA level ([Jhuang et al. 2017] and this work). Dmr1p orthologs from *Candida glabrata* and more distant yeasts species are completely incapable of supporting respiratory growth in the context of *S. cerevisiae* mtDNA, indicating a complete loss of compatibility. These results show good correlation with the presence of the (AUA)_4_AU motif in the 15S rRNA sequence. In all the species where a complete (*S. paradoxus, S. mikatae*, and *S. kudriavzevii*) or partial (*S. bayanus*) compatibility is preserved, at least one such motif can be found, while it is absent from the 15S rRNA sequences in the more distant species, where no compatibility is retained. Remarkably, in silico analysis shows that (AUA)_4_AU is the only 14 nt motif in the 15S rRNA sequences of the analyzed yeast species that shows such phylogenetic distribution, further strengthening the evidence for its key role in the recognition of substrate by the Dmr1 protein. The number of the (AUA)_4_AU motifs is not, however, strictly correlated with the degree of compatibility, as only one such motif can be found in *S. paradoxus*, and in *S. mikatae* that show complete compatibility, as well as in *S. bayanus*, where the compatibility is only partial.

The AUA repeats of at least 14 nt are not limited to the 15S rRNA sequence and can be found in multiple other sites of the yeast mitochondrial genome, including transcribed regions, mostly in the 5′ UTRs and spacer regions of primary transcripts. An interesting exception is the *VAR1* mRNA, which contains multiple AUA repeats. While it is tempting to speculate that Dmr1p might have an additional function in processing and/or stabilizing these RNAs, no experimental evidence points to such involvement. Notably, in *S. cerevisiae* strains where the native *DMR1* gene is replaced with the partially incompatible ortholog from *S. bayanus*, 15S rRNA is the only transcript that is affected, and neither the steady state levels nor processing of other RNAs are perceptibly changed (Jhuang et al. 2017). A more plausible explanation would be that in vivo the interaction of Dmr1p with 15S rRNA is probably achieved through augmentation by RNA secondary structure and/or interactions with other proteins. Even though Dmr1p is not an integral ribosomal component, it was found in the peripheral MIOREX complex—a large cluster of protein factors that copurify with the mitoribosome at low ionic strength (Kehrein et al. 2015). Also, as in yeast mitochondria the levels of rRNAs are orders of magnitude higher than those of other transcripts (Turk et al. 2013), the entire pool of Dmr1p can be bound to 15S rRNA regardless of the presence of potential binding sites elsewhere in the transcriptome. Likewise, preserved compatibility in spite of the presence of only one (AUA)_4_AU motif in *S. paradoxus* and *S. mikatae* also suggests that these motifs are not the sole determinants of the strength of 15S rRNA-Dmr1p interaction. Additionally, suppressor mutations that restore the compatibility between *S. bayanus* Dmr1p and *S. cerevisiae* mtDNA, occur in the regions of 15S rRNA that show no differences between the two species (Jhuang et al. 2017), which suggests that these regions somehow influence the affinity of the protein to its target RNA even though they are not determinants of the specificity of interaction.

The mechanisms of RNA substrate recognition by non-plant PPR proteins appear to be less predictable than the simple modular recognition code proposed for plants. The colinear relation between the PPR motifs and RNA bases is present, if at all, in a rudimentary form. In the case of Dmr1p, the repetitive AUA triplets in the recognized RNA sequence correspond to conserved aspartate and asparagine residues, at positions 1 and 6 of every third PPR motif (the stretch containing this repetitive pattern encompasses 15 motifs in the center of Dmr1p sequence). In plants aspartate in this position (D_1_) determines affinity either for guanine or uridine, in cooperation with amino acid in position 6 of a preceding PPR motif. On the other hand asparagine 6 (N_6_) indicates an affinity for pyrimidine (uridine or cytosine) (Barkan et al. 2012; Yagi et al. 2013; Gully et al. 2015). In our in vitro assays the substrate containing repetitions of UA and AU dinucleotide was not bound by Dmr1p, suggesting that the presence of uridine at every third position, as opposed to every second position, is important for the interaction. It is thus possible that the specificity of RNA substrate recognition by yeast PPR proteins is based on the rhythm of simple repeat sequences. As the yeast mtDNA sequence is remarkably AT-rich, this recognition could simply involve recognizing either the regularity of purines and pyrimidines in the repeats (thus position 6 of each motif would play a crucial role) or the presence of amino and keto group residues (with binding determined by the amino acid in position 1), with secondary structure and interactions with other proteins providing additional layers of specificity. One could even speculate that this mechanism represents a very old and primitive form of PPR protein specificity determination, that operated in the ancestor of plants and Opisthokonta, from which the more precise and versatile plant PPR code evolved. As the present study is the first example of determining the exact sequence recognized by a PPR protein in Opisthokonta, any conclusions regarding the universality of the proposed mechanisms remain pure speculation, until more data for other protein/RNA pairs become available.

## MATERIALS AND METHODS

### Protein expression and purification

Cloning, expression, and purification of the Dmr1-MBP-His_6_ fusion protein was preformed essentially as described previously (Puchta et al. 2010). For purification of the Dmr1-His_6_ fusion protein, the codon-optimized (for yeast) sequence of *DMR1* ORF (without the 26 amino acid amino-terminal mitochondrial targeting sequence) was obtained by custom gene synthesis (Generay Biotech Co.) and cloned in the pET28a vector (Novagen). The pET28a::DMR1 vector was transformed into BL21(DE3) CodonPlus-RIL *E. coli* (Agilent). An amount of 1 mL of the overnight culture was inoculated into 500 mL of AIM (auto induction medium, Formedium), and the cells were grown at 23°C for 36 h. Metal affinity and size exclusion protein purification procedures were performed as described previously (Malecki et al. 2008). The MBP-His_6_ protein used as the negative control was expressed from the pMM41 plasmid (Mayekar et al. 2013) and purified using the same protocol. Purity and composition of the protein preparations was assessed using LC–MS–MS/MS mass spectrometry on the Orbitrap (Thermo Fisher) spectrometer at the Laboratory of Mass Spectrometry, IBB PAS. The purified proteins were stored in a buffer containing 0.5 M NaCl, 50 mM Tris-HCl pH 8, 20% glycerol.

### In vitro RNA synthesis

The sequence of full-length mature 15S rRNA was amplified from yeast genomic DNA using primers 15S_F and 15S_R (all primer sequences are provided in the Supplemental Table S1), and Phusion DNA polymerase (Thermo Fisher), and cloned into pTZ19R (Thermo Fisher). The pTZ19::15S vector was linearized with *Sma*I (Thermo Fisher) and transcribed using the T7 transcription kit (Thermo Fisher), according to the manufacturer's instructions, with α-^32^P-UTP (Hartmann Analytic) added to the reaction to produce radiolabeled substrate, followed by digestion with RNase-free DNase I (Roche). Templates for shorter RNAs were generated by annealing appropriate complementary synthetic DNA oligonucleotides (all sequences are provided in the Supplemental Table S1), containing the T7 promoter sequence, and transcribed in vitro as described above. The RNA transcripts were then purified by electrophoresis in denaturing acrylamide gels (5%–12%, depending on the transcript length) and isolation of the appropriate bands as described previously (Malecki et al. 2008).

### Protein RNA coprecipitation and slot-blot hybridization

In vitro transcription on the linearized pTZ19::15S template was performed as described above in double the usual volume (60 µL) with double amounts of all the reagents. The product was precipitated with EtOH and resuspended in 200 µL RNase free water. The specific activity of this preparation was about 6000 cps/µL. Radioactive RNA was fragmented with NEBNext Magnesium RNA Fragmentation Module (#E6150S) for 7 min. The reaction was stopped by addition of Tris-HCl to 300 mM. Fragmentation of RNA was confirmed by agarose gel electrophoresis.

One third of the fragmented RNA (80 µL) was incubated with 60 µg of the purified protein and 200 ng of unlabeled 15S rRNA competitor in 150 µL total volume of binding buffer (30 mM Tris-HCl pH8, 0.14 M KCl, 2% glycerol, 2 mM DTT, 1 mM spermidine) for 20 min. on ice. Cross-links were induced by UV irradiation using the Hoefer UVC500 device (2′20″ at 3.5 cm from the lamp in an Eppendorf tube). The cross-linked sample was then mixed with 500 µL of buffer A (0.3 M KCl, 50 mM Tris-HCl pH 8, 10% glycerol, 1 mM DTT) and 80 µL of HisPur cobalt resin (Thermo Fisher) equilibrated with buffer A, and incubated on a rotary shaker at 6°C for 1 h. The supernatant was then collected and saved. The resin was washed three times with 400 µL of buffer A (decrease of radioactivity in each subsequent wash was monitored using a Geiger counter), and all the washes were pooled with the original supernatant to yield the unbound fraction. An amount of 400 µL of elution buffer (buffer A + 250 mM imidazole) was then added to the resin and incubated on a rotary shaker for 15 min. The eluate constituted the bound fraction (its radioactivity was confirmed to be at least five times that of the last wash). An amount of 10 µL (>6 U) of proteinase K (Thermo Fisher) and SDS to 0.5% were added to both the bound and unbound fraction preparations, and incubated at 37°C for 30 min. Bound and unbound fraction RNA was then purified by phenol:chlorophorm extraction, followed by EtOH precipitation.

The slot-blot macro array was prepared using 27 synthetic oligonucleotides, 80 nt each, covering the entire sequence of mature 15S rRNA (all oligonucleotide sequences are provided in the Supplemental Table S1), with each probe sequence overlapping the next by 20 nt. An unrelated oligonucleotide, complementary to the *araA* gene from *Aspergillus nidulans* was used as the negative control (N). The array was prepared on Nytran N nylon membrane (Whatman) using the Bio-Dot SF (BioRad) device. 5× SSPE buffer was used to preincubate the membrane and prewash the wells. An amount of 5 µg of each oligonucleotide in 200 µL of 5× SSPE was passed through each well using a vacuum, followed by washing with 500 µL of 5× SSPE. The membrane was then dried, and the oligonucleotides were UV-fixed to the membrane using the Hoefer UVC500 device at 1200 µJ/cm^2^. The membrane was prehybridized for 6–8 h at 42°C in PerfectHyb Plus (Sigma Aldrich). Radioactive RNA samples (bound or unbound) were added to the membrane in 1 mL of PerfectHyb Plus and hybridized overnight at 40°C. The membranes were then washed twice in 2× SSC, 0.1% SDS at room temperature, followed by 0.2× SSC, 0.1% SDS at room temperature and 0.2× SSC, 0.1% SDS at 37°C. Autoradiography of the membrane was performed for at least 48 h on a phosphor screen (Fuji). Typhoon FLA 9000 laser imaging system (GE Healthcare) device was used to quantitate the signal from each slot, and the ratio of bound/unbound fraction signal for each probe was then calculated. The experiment was performed in triplicate.

### Electrophoretic mobility shift assays (EMSA)

The RNA fragments of 14–35 nt were produced by in vitro transcription of templates obtained by annealing pairs of complementary synthetic oligonucleotides. About 30–60 cps of radiolabeled RNA was used in a single binding reaction. Binding and the electrophoretic mobility shift assays were performed as described previously (Puchta et al. 2010), with 0.2 µg of unlabeled bacterial tRNA (Roche) added to each sample as a nonspecific competitor. In the case of 80 nt fragments, 100 ng of unlabeled 80 nt fragments of identical sequence to the respective labeled oligonucleotides was used as a specific competitor. The total protein concentration was kept constant with the addition of BSA, which was also used as the negative control. In every case, each sample had the final volume of 15 µL.

### RNase protection assays

#### Terminator 5′-phosphate-dependent exonuclease

Gel-purified radiolabeled RNA fragments obtained using in vitro transcription were treated with RNA 5′ Polyphosphatase (Epicentre) according to the manufacturer's protocol, purified by phenol:chlorophorm extraction and EtOH precipitation, and resuspended in RNase free water. Radiolabeled RNA (30–40 cps) was incubated with 1.2 µg of Dmr1-His_6_ fusion protein purified by metal affinity and size exclusion or with 1.2 µg of BSA (negative control) in 14 µL of reaction buffer (50 mM Tris-HCl pH 7.5, 100 mM NaCl, 4 mM DTT, 2 mM MgCl_2_, 2 mM sodium phosphate pH 7.0) with 1 U/μL of RiboLock RNase inhibitor (Thermo Fisher) for 20 min. on ice. 0.5 U of Terminator 5′-Phosphate-Dependent Exonuclease (Epicentre) was then added, and the reaction incubated for 30 min at 30°C.

#### Polynucleotide phosphorylase (PNPase)

Gel-purified radiolabeled RNA fragments obtained using in vitro transcription (30–40 cps) were incubated with 1.2 µg of Dmr1-His_6_ fusion protein purified by metal affinity and size exclusion or with 1.2 µg of BSA (negative control) in 14 µL of reaction buffer (100 mM KCl, 2 mM DTT, 20 mM Tris-HCl pH 8.0, 10% glycerol, 1 mM spermidine, 2 mM MgCl_2_, 2 mM sodium phosphate pH 7.0) with 1 U/μL of RiboLock RNase inhibitor (Thermo Fisher) for 20 min on ice. An amount of 0.2 µg (>0.1 U) U of recombinant *Synechocystis sp.* PNPase (Sigma) was then added, and the reaction incubated for 30 min at 25°C.

#### Yeast Dis3p exoribonuclease

Gel-purified radiolabeled RNA fragments obtained using in vitro transcription (30–40 cps) were incubated with 0.8 µg of Dmr1-His_6_ fusion protein purified by metal affinity and size exclusion or with 0.8 µg of BSA (negative control) in 14 µL of reaction buffer (50 mM Tris-HCl pH 7.5, 100 mM NaCl, 4 mM DTT, 2 mM MgCl_2_) with 1 U/μL of RiboLock RNase inhibitor (Thermo Fisher) for 20 min on ice. 0.3 µg of purified Dis3p (a gift from Dr. Rafał Tomecki) was then added, and the reaction incubated for 30 min at 30°C.

In each assay the reaction was stopped by addition of the same volume of the electrophoresis loading buffer (95% formamide, 0.025% SDS, 0.025% bromophenol blue, 0.025% xylene cyanol, 0.5 mM EDTA), and the products were separated in a 20% denaturing polyacrylamide gel.

### Molecular cloning and yeast genetics

Standard yeast genetic media and methods were as described previously (Dujardin et al. 1980; Burke et al. 2000). Yeasts were transformed using the LiAc/PEG/ssDNA protocol (Gietz and Woods 2002). Construction of a plasmid containing the *S. cerevisiae DMR1* gene in the YCplac111 (ARS-CEN, *LEU2*) vector was described previously (Lipinski et al. 2011). The *DMR1* ORF in this vector was replaced by orthologous sequences from *S. paradoxus*, *S. bayanus*, *C. glabrata, Kluyveromyces lactis, Debaryomyces hansenii*, and *Yarrowia lipolytica* using overlap extension PCR (Bryksin and Matsumura 2010) (all primer sequences are provided in the Supplemental Table S1). The nonstandard CTG (serine) codon in the *D. hansenii* gene was changed to the standard TCG (serine) using site-directed mutagenesis following the Quikchange Site Directed Mutagenesis (Agilent) PCR protocol. All the constructs were verified by Sanger sequencing performed in the Laboratory of DNA Sequencing PAS.

Construction of the HS-DPPR strain with the *DMR1* gene deleted from the genome, and carrying a copy on a YEplac195 (2µ, *URA3*) vector (*MAT* α*, ade2; trp1; ura3; leu2; his3; dmr1::kanMX4*; [rho^+^ intronless]; [pDMR1-2μ]) was described previously (Lipinski et al. 2011). Vectors carrying cloned *DMR1* orthologs were introduced into this strain by transformation, and the *URA3* maintenance vector was removed by 5-FOA counter-selection (Sikorski and Boeke 1991). For testing strains were grown overnight in complete synthetic medium (CSM) without leucine. Starting with OD_600_ = 1 of the preculture, a series of 10-fold dilutions were spotted on YPD (glucose) and YPG (glycerol) plates and incubated at 30°C for 3 d. Empty YCplac111 vector and the vector carrying the *S. cerevisiae DMR1* sequence were used as negative and positive controls, respectively.

### In silico methods

The I-TASSER server (Yang and Zhang 2015) was used to obtain the model of Dmr1p structure, which was visualized and analyzed using the UCSF Chimera software (Pettersen et al. 2004). TPRpred (Karpenahalli et al. 2007; Zimmermann et al. 2018) was used to identify the PPR motifs, along with structure based analysis and previous results (Lipinski et al. 2011). PPR motif conservation profiles were generated using WebLogo (Crooks et al. 2004). The GenBank accession numbers of the entries used to retrieve the 15S rRNA sequences were as follows: *S. cerevisiae*: KP263414.1, *S. paradoxus*: AF114922.1, *S. bayanus*: AF114901.1, *C. glabrata*: AJ511533.1, *K. lactis* : AY654900.1, *Y. lipolytica*: AJ307410.1, *D. hansenii*: DQ508940.1, *S. mikatae*: KX657747.1, *S. kudriavzevii*: KX657746.1, *S. castellii*: AF437291.1, *L. kluyveri*: HE664112.1, *K. waltii*: AF442341.1, *A. gossypii*: AE016821.1, *C. albicans*: AF285261.1. The sequences and the Python scripts used to analyze them are available at https://github.com/golikp/dmr1_motif. The cladogram in Figure 4A is based on the published fungal maximum likelihood phylogeny reconstructed using a concatenated alignment of 153 universally distributed fungal genes (Fitzpatrick et al. 2006).

## SUPPLEMENTAL MATERIAL

Supplemental material is available for this article.

## Supplementary Material

Supplemental Material
