# Supplementary material for: Yeast pentatricopeptide protein Dmr1 (Ccm1) binds a repetitive AU-rich motif in the small subunit mitochondrial ribosomal RNA
Source: RNA. 2020 Sep;26(9):1268–82. doi: 10.1261/rna.074880.120 (PMC7430664; doi:10.1261/rna.074880.120)
Supplement: Supplemental Material [file supp_26_9_1268__index.html]

Yeast pentatricopeptide protein Dmr1 (Ccm1) binds a repetitive AU-rich motif in the small subunit mitochondrial ribosomal RNA — Supplemental Material 

# Yeast pentatricopeptide protein Dmr1 (Ccm1) binds a repetitive AU-rich motif in the small subunit mitochondrial ribosomal RNA

## Supplemental Material

- Supplemental\_table\_S1.docx
- Supplemental\_table\_S2.docx
