# Supplementary material for: Yeast pentatricopeptide protein Dmr1 (Ccm1) binds a repetitive AU-rich motif in the small subunit mitochondrial ribosomal RNA
Source: RNA. 2020 Sep;26(9):1268–82. doi: 10.1261/rna.074880.120 (PMC7430664; doi:10.1261/rna.074880.120)
Supplement: Supplemental Material [file supp_074880.120_Supplemental_table_S1.docx]

**Supplementary table S1:** Sequences of synthetic oligonucleotide probes and primers

| **Name** | **Sequence (5’-3’)** | **Experiment** |
| --- | --- | --- |
| 15S_F | GGGAAGCTTAATTTATAAGAATATGATGTTGGTTCAG | Amplification of *S. cerevisiae* 15S rRNA |
| 15S_R | GGGCTGCAGTGTAAGAATATTTAAGATATTTATAAGCCCAC |  |
| 7_24_F | TAATACGACTCACTATAGGTTATAATATGGGTAATAGACGTGC | *In vitro* transcription^a^ |
| 7_24_REV | GCACGTCTATTACCCATATTATAACCTATAGTGAGTCGTATTA |  |
| 15_24_F | TAATACGACTCACTATAGGAAATAATAATAATAATTATAAGAC |  |
| 15_24_REV | GTCTTATAATTATTATTATTATTTCCTATAGTGAGTCGTATTA |  |
| 20_24_F | TAATACGACTCACTATAGGTTATAATAATAATAATAATAATAT |  |
| 20_24_REV | ATATTATTATTATTATTATTATAACCTATAGTGAGTCGTATTA |  |
| N_24_F | TAATACGACTCACTATAGGAAAAGGATATATATATAATATATA |  |
| N_24_REV | TATATATTATATATATATCCTTTTCCTATAGTGAGTCGTATTA |  |
| 7_14_F | TAATACGACTCACTATAGGATAATATGGGTAAT |  |
| 7_14_REV | ATTACCCATATTATCCTATAGTGAGTCGTATTA |  |
| 15_14_F | TAATACGACTCACTATAGGATAATAATAATAAT |  |
| 15_14_REV | ATTATTATTATTATCCTATAGTGAGTCGTATTA |  |
| N_14_F | TAATACGACTCACTATAGGTATATATATAATAT |  |
| N_14_REV | ATATTATATATATACCTATAGTGAGTCGTATTA |  |
| T7_prom^b^ | GGGGTAATACGACTCACTATAGG |  |
| 15_35_REV^b^ | ATTAGTCTTATAATTATTATTATTATTTCATCTTTCCTATAGTGAGTCGTATTACCCC |  |
| 6_F^c^ | GGCTAATACGACTCACTATAGGATAGACGTGCTATAATAAAATG |  |
| 6_R^c^ | ATCGAATTTCAGATTATAATTC |  |
| 7_F^c^ | GGCTAATACGACTCACTATAGGATTTATTAATATAAAGAAAGG |  |
| 7_R^c^ | ATATATTTTATATAATTTTATTATC |  |
| 11_F^c^ | GGCTAATACGACTCACTATAGGTGAAGAGTACGTTAGCAATAATG |  |
| 11_R^c^ | TTATAATATTCAAAATATGGTAAGG |  |
| 15_F^c^ | GGCTAATACGACTCACTATAGGATTATATATTATAATTTAGAG |  |
| 15_R^c^ | GTTTTAATCCCTCAATGTCAG |  |
| 17_F^c^ | GGCTAATACGACTCACTATAGGTTAATAATATATTTTAATAGTCC |  |
| 17_R^c^ | TATAATATATAATTCATTCTACGG |  |
| 19_F^c^ | GGCTAATACGACTCACTATAGGTAAATTGATTAAAAATAAAATCC |  |
| 19_R^c^ | GGCACAAATATTAGTCAGGAC |  |
| 20_F^c^ | GGCTAATACGACTCACTATAGGGATCCAGTTACTTATTAGGATG |  |
| 20_R^c^ | CATTATTTTAATTATTTATGG |  |
| 24_F^c^ | GGCTAATACGACTCACTATAGGTATAATAAAAAGGATATATATAT |  |
| 24_R^c^ | GGATCGTTGGCTAGGTTTAACTC |  |
| #1 | TGTAAGAATATTTAAGATATTTATAAGCCCACCGCAGGTTCCCCTACGGTAACTGTATTTCAACTTCGCATTAATTCATA | Macro array hybridisation probe |
| #2 | CAACTTCGCATTAATTCATATTATTTCTGATATAAATAAATATAAATTAATAATTATTAATATTTATTAAAAAATATTAT |  |
| #3 | TATTTATTAAAAAATATTATATAAATAATAAGATAATAATATGTTTCAACGCGTGATGAGTGATTAGTGCGAAACAGTTA |  |
| #4 | TGATTAGTGCGAAACAGTTAGAATATTCACCGTAACATACTAATTTACGTATTACTAGCAATTCTTTTTTCATATAATCG |  |
| #5 | ATTCTTTTTTCATATAATCGAATTTCAGATTATAATTCATATTAATATATAATAAAAAAATATATTAAAAATTAAAATGT |  |
| #6 | TATATTAAAAATTAAAATGTTTTATATTATTAATTAAATATAATTAAATATATTTTATATAATTTTATTATCATTTTATT |  |
| #7 | AATTTTATTATCATTTTATTATAGCACGTCTATTACCCATATTATAAGGATCATTATGATTTGTCTTAATTCCTTTCTTT |  |
| #8 | TTGTCTTAATTCCTTTCTTTATATTAATAAATAATATAAAATTATATATAATTAAAATTATATATATGGAGTTTTGTTCG |  |
| #9 | TATATATGGAGTTTTGTTCGTTTATGAACTTAATCTAAAACTTTGCAGCACGAACTAAAGACAACAATGTAACGCCTGTA |  |
| #10 | ACAACAATGTAACGCCTGTAATATAATAATTATAATAATTATTATAATATTCAAAATATGGTAAGGTTAGTCGTGGATTA |  |
| #11 | GTAAGGTTAGTCGTGGATTATCGAATTAAATAACATGCTCCACTGCTTAAGTCTGTAACCGTCTATTGTTTTGAGTTTCA |  |
| #12 | GTCTATTGTTTTGAGTTTCATTATTGCTAACGTACTCTTCAGGTGGAATACTTTCATTTTCATTTATTATTTATATATAA |  |
| #13 | CATTTATTATTTATATATAATATATATTATAAATAATTGTATTCATAGTTTACTACTAGAACTACACGGGTATCGAATCC |  |
| #14 | ACTACACGGGTATCGAATCCGTTTCGCTACTCTAGTTTTAATCCCTCAATGTCAGTTAATATTTAATTAATATTTTCACA |  |
| #15 | ATTTAATTAATATTTTCACATATATTAGTCTTATAATTATTATTATTATTTCATCTTTACTATTATAATTCTTTAATTAT |  |
| #16 | TATTATAATTCTTTAATTATATTTTATTAACTCTAAATTATAATATATAATTCATTCTACGGATCCTTTAAACCATTATG |  |
| #17 | GGATCCTTTAAACCATTATGATTAACGCTCGCCCTCTTTGTGTTACCGCGACTGCTGGCACAAATATTAGTCAGGACTAT |  |
| #18 | CAAATATTAGTCAGGACTATTAAAATATATTATTAATAATAATAAAATTAATATTATTATTAATATATATATCCATATAA |  |
| #19 | TAATATATATATCCATATAAAAATATATATGGTAATTAATATCATTATTTTAATTATTTATGGATTTTATTTTTAATCAA |  |
| #20 | TGGATTTTATTTTTAATCAATTTATATATATATATTATTATTATTATTATTATAAATATTTAATATTTATAAATAAAATA |  |
| #21 | TAATATTTATAAATAAAATAAAATATTTTTATATATCATCCTAATAAGTAACTGGATCAATCTTTCGATCATTGTCCAAT |  |
| #22 | TCTTTCGATCATTGTCCAATATTCCTCACTGCTGTATCTTATAGATATTGACTATATTTCAGAGTCAACGTGATCGTTCT |  |
| #23 | AGAGTCAACGTGATCGTTCTAACTTTCATTATCGATTATGGATCGTTGGCTAGGTTTAACTCTTAATAAACCTACTACCT |  |
| #24 | TCTTAATAAACCTACTACCTAAACCATTATTGGCTTGACTATAGATAAATATATATTATATATATATCCTTTTTATTATA |  |
| #25 | TATATATCCTTTTTATTATATAAATTATTTTTATATAATAATATATTAAGTATTTAGCTTATTCTATAGTTCATTAATTC |  |
| #26 | ATTCTATAGTTCATTAATTCCTAATAAAATTACTCACGTTTACACCACATATTTATTATCTTATCAATAATAAACGTATG |  |
| #27 | TTATCAATAATAAACGTATGATTCGCATGTGTCATGTCCTTATTTAGCGCTTAATCTGAACCAACATCATATTCTTATAAATTTTTTAC |  |
| N | GATAACCCGACAATCTTCATAAATCATTATTCTTGTCTAGCTCTGTTAATGAAATCGCTGACTGACGTAATTACA | Macro array hybridisation probe – negative control |
| Spar_F | GCATTGTTTCACAAGTCCTAACAGATAGATAAATATAAACATGTACATGGCCAGATGTGGCCC | Amplification and cloning of *S. paradoxus DMR1* |
| Spar_R | GTATATGTCGTTTCTTGATATATAATCACTTTTAGTCTAATGATGACTACATCTTAAGTTCTTGCTCC |  |
| Sbay_F | GCATTGTTTCACAAGTCCTAACAGATAGATAAATATAAACATGTACATGGTCAGATGTGGCCTC | Amplification and cloning of *S. bayanus DMR1* |
| Sbay_R | GTATATGTCGTTTCTTGATATATAATCACTTTTAGTCTAATGATTAAAATTTGAGCTCCTGCTCTTC |  |
| Cgla_F | CACAGGAAAATAGCATTGTTTCACAAGTCCTAACAGATAGATAAATATAAACATGCGAACTGTACTACCAGTTCACAGG | Amplification and cloning of *C. glabrata DMR1* |
| Cgla_R | GGACGCGTATATGTCGTTTCTTGATATATAATCACTTTTAGTCTAATGATTAGGATAACTTGAGTTCCTCGATTTGTTC |  |
| Klac_F | GCATTGTTTCACAAGTCCTAACAGATAGATAAATATAAACATGCTTTCACTTGGAAAGAATGGTAAGACTTCG | Amplification and cloning of *K. lactis DMR1* |
| Klac_R | GTATATGTCGTTTCTTGATATATAATCACTTTTAGTCTAATGATCAAAGGTCTAATGACTTGATCTCTTCCTCCAATTC |  |
| Dhan_F | GCATTGTTTCACAAGTCCTAACAGATAGATAAATATAAACATGATGTTAAGGCTCTCTGTAAAAAAGAGATGC | Amplification and cloning of *D. hansenii DMR1* |
| Dhan_R | GTATATGTCGTTTCTTGATATATAATCACTTTTAGTCTAATGATTAATATCCACGTTCCATGACGTACCTC |  |
| Ylip_F | GCATTGTTTCACAAGTCCTAACAGATAGATAAATATAAACATGCTCAGAGCACGCCTTCTAGTGCC | Amplification and cloning of *Y. lipolytica DMR1* |
| Ylip_R | GTATATGTCGTTTCTTGATATATAATCACTTTTAGTCTAATGATCAAATGCTGAAAACGTCAAATTCAGCTGATTTTTCC |  |
| Dhan_mutF | GTTTAATCCGTCGTTTGTCAATACTGACTGCGC | CTG->TCG correction of *D. hansenii DMR1* |
| Dhan_mutR | GTATTGACAAACGACGGATTAAACATTAAAGTGTG |  |
| del1F | GAATTTTCTGTTTGGTTAAATGGTAC | Generation of deletion variants by PCR |
| del2R | AAATCTTGCATAAGCAAGCATC |  |
| del2_4F | TAATAACCCAGCTTTCTTGTACAAAG |  |
| del3F | GTCTTCAATAAAATTTCACTACAC |  |
| del4R | ATGCAAATTCCTAACCATGGAATAAG |  |

^a^ In each *in vitro* transcription reaction the _F and _REV oligonucleotides were annealed to form the double-stranded template. The TAATACGACTCACTATAGG T7 promoter sequence can be found appended to each oligonucleotide.

^b^ A partially double-stranded template obtained by annealing the T7_prom and 15_35_REV oligonucleotides was used to produce the 15_35 RNA substrate.

^c^These oligonucleotides were used in PCR reactions, in order to obtain templates for in vitro transcription used to produce 80 nt long substrates for EMSA analysis
