# Supplementary material for: Yeast pentatricopeptide protein Dmr1 (Ccm1) binds a repetitive AU-rich motif in the small subunit mitochondrial ribosomal RNA
Source: RNA. 2020 Sep;26(9):1268–82. doi: 10.1261/rna.074880.120 (PMC7430664; doi:10.1261/rna.074880.120)
Supplement: Supplemental Material [file supp_074880.120_Supplemental_table_S2.docx]

**Supplementary Table S2** Peptides identified in the Dmr1p-MBP-His_6_ fusion protein preparation by mass spectrometry.

| ***E. coli*** | |
| --- | --- |
| **Acc. no.** | **Description** |
| gi\|15799694 | dnaK gene product [Escherichia coli O157:H7 str. EDL933] |
| gi\|147989 | trigger factor [Escherichia coli] |
| gi\|15804576 | rplL gene product [Escherichia coli O157:H7 str. EDL933] |
| gi\|15804153 | secB gene product [Escherichia coli O157:H7 str. EDL933] |
| gi\|15800202 | htpG gene product [Escherichia coli O157:H7 str. EDL933] |
| gi\|15804823 | yjfG gene product [Escherichia coli O157:H7 str. EDL933] |
| gi\|15799695 | dnaJ gene product [Escherichia coli O157:H7 str. EDL933] |
| gi\|993029 | glutaredoxin‑like protein, partial [Escherichia coli] |
| gi\|15803210 | csrA gene product [Escherichia coli O157:H7 str. EDL933] |
| gi\|930016 | monomeric lac repressor [Escherichia coli] |
| gi\|15802713 | yeiE gene product [Escherichia coli O157:H7 str. EDL933] |
| gi\|157154864 | hydroperoxidase II [Escherichia coli E24377A] |
| gi\|1421648 | Chain A, Conformational Variability In The Refined Structure Of The Chaperonin Groel At 2.8 Angstrom Resolution |
| gi\|218555114 | hypothetical protein ECIAI1_2644 [Escherichia coli IAI1] |
| gi\|388394567 | Tn7‑like transposition protein TnsC [Escherichia coli 541‑15] |
| gi\|315618747 | HAD‑superfamily hydrolase, subfamily IA, variant 3 family protein [Escherichia coli 3431] |
| gi\|377994839 | phage‑related family protein [Escherichia coli DEC6B] |
| gi\|41114 | analogue of ATP‑dependent protease regulatory subunit [Escherichia coli K‑12] |
| gi\|191169364 | gp18 [Escherichia coli B7A] |
| gi\|15803862 | slyD gene product [Escherichia coli O157:H7 str. EDL933] |
| ***S. cerevisiae*** | |
| **Acc. no.** | **Description** |
| gi\|6321589 | Ccm1p (Dmr1p) [Saccharomyces cerevisiae S288c] |
| gi\|172713 | 70kDal heat shock protein, partial [Saccharomyces cerevisiae] |
| gi\|6322329 | hypothetical protein YJL132W [Saccharomyces cerevisiae S288c] |
| gi\|323303470 | Rsn1p [Saccharomyces cerevisiae FostersB] |
| gi\|197724939 | Chain A, Crystal Structure Of Rtt109 |
| gi\|315113350 | Chain S, Localization Of The Large Subunit Ribosomal Proteins Into A 6.1 A Cryo‑Em map of Saccharomyces cerevisiae Translating 80s Ribosome |
